# Efficacy of a behavior change program on cardiovascular parameters in patients with hypertension: a randomized controlled trial

**DOI:** 10.31744/einstein_journal/2020AO5227

**Published:** 2020-01-27

**Authors:** Aline Mendes Gerage, Tânia Rosane Bertoldo Benedetti, Bruno Remígio Cavalcante, Breno Quintella Farah, Raphael Mendes Ritti-Dias

**Affiliations:** 1 Universidade Federal de Santa Catarina FlorianópolisSC Brazil Universidade Federal de Santa Catarina, Florianópolis, SC, Brazil.; 2 Universidade de Pernambuco RecifePE Brazil Universidade de Pernambuco, Recife, PE, Brazil.; 3 Universidade Federal Rural de Pernambuco RecifePE Brazil Universidade Federal Rural de Pernambuco, Recife, PE, Brazil.; 4 Universidade Nove de Julho São PauloSP Brazil Universidade Nove de Julho, São Paulo, SP, Brazil.

**Keywords:** Hypertension, Blood pressure, Cardiac rehabilitation, Lifestyle, Health behavior

## Abstract

**Objective:**

To investigate the efficacy of a behavior change program named *Vida Ativa Melhorando a Saúde* on cardiovascular parameters in hypertensive patients.

**Methods:**

Ninety hypertensive patients aged over 40 years were randomly allocated to one of two groups: *Vida Ativa Melhorando a Saúde* or Control (n=45 respectively). Patients in the *Vida Ativa Melhorando a Saúde* group took part in a behavior change program aimed to encourage changes in physical activity levels and eating habits, according to the Social Cognitive Theory. The program consisted of 90-minute weekly group meetings conducted by a physical therapist and a dietitian. One chapter of the educational material (workbook) provided was discussed per meeting. Participants in the Control Group attended a single educative lecture on lifestyle changes. Brachial and central blood pressure, arterial stiffness and endothelial function parameters were measured pre- and post-intervention.

**Results:**

*Vida Ativa Melhorando a Saúde* led to reduction of brachial (131.3±15.8mmHg to 125.1±17.3mmHg; p<0.01) and central (123.6±16.3mmHg to 119.0±20.6mmHg; p=0.02) systolic and brachial diastolic (123.6±16.3mmHg to 119.0±20.6mmHg; p<0.01) blood pressure values, and improvement of post-occlusive reactive hyperemia (from 5.7±2.5mL·100mL^−1^ to 6.5±2.1mL·100mL^−1^ tissue·min^−1;^ p=0.04). No changes in body composition, heart rate and arterial stiffness parameters were detected in both groups (p>0.05).

**Conclusion:**

*Vida Ativa Melhorando a Saúde* program improved blood pressure and microvascular reactivity in hypertensive patients. Trial registration: ClinicalTrials.gov: NCT02257268

## INTRODUCTION

Hypertension is a leading cause of cardiovascular disease^1^ and is highly associated with obesity,^2^ increased arterial stiffness^3^ and reduced microvascular reactivity.^4^ Physical inactivity and unhealthy diets are major risk and prognostic factors for hypertension; therefore, physical activity and eating habits are the cornerstone of lifestyle modification approaches aimed at hypertensive patients.^1 , 5 , 6^ Increased levels of physical activity combined with a healthy diet have been shown to benefit cardiovascular health;^7^ still, only 30% of hypertensive patients comply with physical activity recommendations,^8 , 9^ and less than 10% report healthy eating habits.^10^

Lifestyle change programs based on behavioral change theories are thought to be promising strategies to revert this situation. In developed countries, such long-term programs (>6 months) have been shown to reduce systolic and diastolic blood pressure (BP).^11^ However, long-term programs are more difficult to implement in clinical settings in developing countries due to their high cost. Hence the need to understand the effects of short-term behavior change programs promoting patient autonomy and empowerment. In this context, a short-term behavior change program named *Vida Ativa Melhorando a Saúde* (VAMOS) and aimed to promote physical activity and healthy eating habits among adult and elderly individuals has been recently developed in Brazil.^12^ The effectiveness of this program in maintaining physical activity, and improving eating habits and quality of life in hypertensive patients, has been demonstrated in a previous study by the same research group.^13^

## OBJECTIVE

To analyze the efficacy of *Vida Ativa Melhorando a Saúde* program on cardiovascular parameters in patients with primary hypertension.

## METHODS

### Study participants

This non-pharmacological, randomized controlled trial was conducted with 90 volunteers at *Universidade de Pernambuco* , Recife (PE), Brazil, in 2014. Recruitment strategies have been described elsewhere.^14^

Inclusion criteria were as follows: age over 40 years, diagnosis of hypertension,^15^ and use of antihypertensive drugs for at least 3 months prior to the study. Diabetic patients, patients with a previous diagnosis of cardiovascular disease ( *e.g* ., ischemic heart disease, heart failure, coronary artery disease, peripheral arterial disease or stroke), or with physical disabilities, or involved in regular physical activity programs were not included. Changes in antihypertensive drug type or dose were exclusion criteria.

### Randomization

Participants were blocked randomized (by a researcher not directly involved in recruitment or data collection) to one of two groups: VAMOS (n=45) and Control (n=45). Randomization was carried out according to sex and pre-intervention BP using a random number table.

### Experimental design

Participants were asked to visit the laboratory twice prior to intervention start (pre-intervention stage). In the first visit, anthropometric, body composition and brachial BP assessments were carried out. In the second visit, scheduled at the same time of day as the first, brachial and central BP, arterial stiffness, basal blood flow and microvascular reactivity were measured.

Control Group participants attended an educative lecture about lifestyle changes, whereas those in the VAMOS Group took part in a 12-week behavioral change program. At the end of the 12-week period, all participants were re-evaluated (post-intervention stage) using the same (pre-intervention) procedures. Researchers in charge of assessments and data analysis were blinded to randomization.

### The behavior change program *Vida Ativa Melhorando a Saúde*

VAMOS Group participants were enrolled in a behavioral program aimed to encourage behavior changes associated with a healthy lifestyle, including physical activity and eating habits, according to the social cognitive theory.^16^ The program consisted of 90-minute weekly group meetings conducted by a physical therapist and scheduled over the course of 12 consecutive weeks. One chapter of the educational material (workbook) was discussed per meeting.^13^ Briefly, each workbook chapter comprised several topics related to physical activity, healthy eating habits and behavior change strategies, such as definition of physical activity and healthy eating habits concepts, assessment of behavior change stages, healthy eating and physical activity routine planning and implementation, overcoming of barriers and challenges, support gathering and progress monitoring ( *e.g* ., pedometers), stress management tips, trust building and revisiting of initial goals. Participants should attend all meetings; when failing to attend (up to 25%), content delivery should be rescheduled.

### Body composition assessment

Total and trunk body fat were estimated using dual-energy X-ray absorptiometry (Lunar Prodigy DXA, model NRL 41990, GE Lunar, Madison, WI), as per manufacturer’s instructions. Percent body fat was calculated by dividing fat content by segment (trunk or whole body) weight.

### Cardiovascular measurements

Patients were instructed to eat a light meal, avoid moderate-to-vigorous physical activity for at least 24 hours, and not smoking or drinking alcohol or caffeine for at least 12 hours before visiting the laboratory for cardiovascular measurements. Measurements were taken after a 10-minute rest period, with patients in the supine position, in a quiet, temperature-controlled environment.

Brachial systolic and diastolic BP were measured in the left arm using an automated oscillometric device (Omron HEM 742-E, Bannockburn, USA). Three measurements were taken per day at 1-minute intervals, on two non-consecutive days.^17^ Mean BP values were used in the analysis. All measurements were made by the same person.

Forearm blood flow (FBF) was measured using venous occlusion plethysmography (Hokanson, EC6, USA), as described elsewhere.^18^ Measurements were taken in the non-dominant forearm with subjects resting in the supine position. A cuff was placed above the hand and inflated to approximately 240mmHg to interrupt forearm blood flow. A second cuff was placed around the arm and inflated to subdiastolic BP (40 to 60mmHg) for 10 seconds, every 20 seconds. A mercury gauge was placed around the widest part of the forearm to detect changes in forearm circumference in response to arterial blood influx. Forearm circumference change slope was used to estimate basal FBF. Post-occlusive reactive hyperemia (PORH) was then assessed. The wrist cuff proximal to the measurement site was inflated to 200mmHg and occlusion was maintained for 3 minutes. The cuff was then deflated and FBF measured for 3 minutes, as previously described. Forearm blood flow and PORH were defined as the mean value of nine measurements taken prior to and after the ischemic period, respectively.

Central BP and arterial stiffness parameters were measured using applanation tonometry (SphygmoCor, AtcorMedical, Sydney, Australia). Central BP values were estimated using a validated transfer function algorithm provided by SphygmoCor^®^ software. Carotid-femoral pulse wave velocity (cfPWV) and augmentation index were used to assess arterial stiffness and wave reflection, respectively. These parameters were measured by the same person, as per guidelines.^19^

### Statistical analysis

Data were stored and analyzed using (SPSS), version 17.0, for Windows software. Data normality and homogeneity of variance were investigated using the Shapiro-Wilk test and the Levene test, respectively. Continuous variables were expressed as mean and standard deviation, and the categorical variables, as relative frequency. Pre-intervention intergroup differences were analyzed using independent *t* test or the χ^2^ test. Two-way analysis of variance (ANOVA) for repeated measures was used for inter- and intragroup comparisons. Whenever the sphericity assumption was violated (Mauchly’s test), analyses were adjusted using Greenhouse-Geisser correction. Whenever the F-ratio was significant, the Fisher’s least significant difference (LSD) *post-hoc* test was employed to identify differences between means. Effect size (ES) was calculated to investigate the magnitude of differences based on Cohen’s d.

Apart from per protocol analysis (participants evaluated at both study time points, and attending at least 75% of behavior change meetings in VAMOS Group, or the lecture, in Control Group), intention-to-treat analysis was also carried out. For this purpose, randomized participants who dropped out the study were invited for post-intervention reassessments. The last observation carried forward (LOCF) approach was used to account for missing data (<10%).

Sample size was calculated using GPower software (3.1.9). Taking systolic BP as the primary outcome, alpha of 95%, power of 80% and ES of 1.19,^20^ the sample size required corresponded to 13 participants per group.

The research protocol was approved by the local Ethics Committee (protocol 711.420; CAAE: 24252513.4.0000.0121) and registered at ClinicalTrials.gov (protocol NCT02257268). An informed consent form was signed by all participants.

## RESULTS

**Figure 1:**
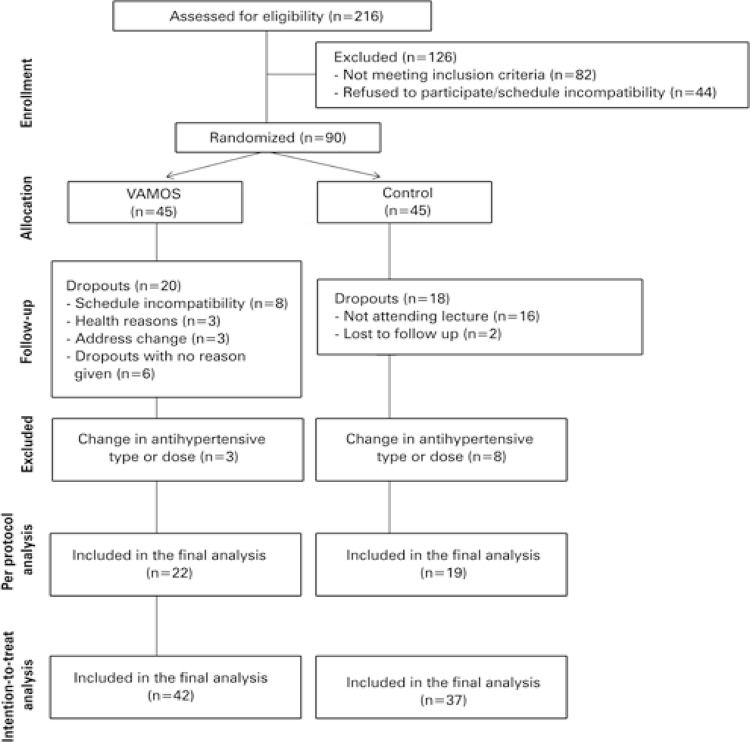
Study flowchart VAMOS: *Vida Ativa Melhorando a Saúde* .

Figure 1 shows the flowchart of study participants. Out of 216 individuals assessed for eligibility, 126 were excluded for not meeting inclusion criteria, refusing to participate, or schedule incompatibility. Of 45 participants randomized to each group, 48.9% of VAMOS (15 women and 7 men) and 42% of Control (14 women and 5 men) Group participants adhered to the 12-week intervention (≥75%), or attended the lecture presented for post-intervention assessments, and did not change their antihypertensive drug type or dose. There were 38 dropouts (20 in VAMOS and 18 in Control Group) and 11 exclusions due to changes in antihypertensive drug type or dose (3 in VAMOS and 8 in Control Group).

General characteristics of both groups at pre-intervention are shown in table 1 .

**Table 1:** General characteristics of participants of groups at pre-intervention stage

Variables	Control (n=19)	VAMOS (n=22)	p value
Female sex, (%)	74	68	0.70
Age, years	57±9	59±10	0.50
Marital status, married, (%)	47	68	0.36
Schooling level, study years	12±4	11±5	0.77
Number of antihypertensives	2.2±0.9	2.1±0.9	0.68
Diuretics, (%)	53	64	0.48
AT1 blockers, (%)	53	59	0.68
Adrenergic inhibitors, (%)	47	36	0.48
Calcium blockers, (%)	37	27	0.52
ACE inhibitors, (%)	26	23	0.79
Renin inhibitor, (%)	5	0	0.28

Results expressed as % or mean±standard deviation.

VAMOS: *Vida Ativa Melhorando a Saúde;* AT1: angiotensin receptors; ACE: angiotensin-converting enzyme.

Demographic characteristics and antihypertensive drugs used did not differ between the VAMOS and the Control Groups at pre-intervention (p<0.05). Most patients in both groups were women, married and using more than one antihypertensive drug, the most common being diuretics and AT1 blockers.

Group *versus* time interactions had no major effects on body composition, suggesting body composition parameters remained unchanged throughout the study in both groups (p>0.05) ( Table 2 ).

**Table 2:** Body composition of participants in both groups at pre and post-intervention stages

Variables	Control (n=19)	VAMOS (n=22)	Effects		

			Group	Time	Interaction
Weight, kg					
Pre	79.2±10.9	81.1±21.1	0.79	0.11	0.54
Post	79.8±11.3	80.8±21.0			
ES	0.05	-0.01			
Body fat, %					
Pre	42.2±8.3	41.4±6.5	0.71	0.37	0.94
Post	41.9±7.9	41.0±6.4			
ES	-0.04	-0.06			
Trunk fat, %					
Pre	43.8±8.2	42.3±6.7	0.62	0.64	0.43
Post	43.7±7.6	42.9±6.8			
ES	-0.01	0.09			
Muscle mass, kg					
Pre	42.0±7.2	44.3±11.1	0.81	0.70	0.10
Post	44.0±7.2	43.1±12.2			
ES	0.28	-0.10			
BMD, g/cm^2^					
Pre	1.1±0.2	1.1±0.1	0.40	0.62	0.15
Post	1.1±0.1	1.1±0.1			
ES	0	0			

Results expressed as mean±standard deviation.

VAMOS: *Vida Ativa Melhorando a Saúde* ; ES: effect size; BMD: body mineral density.

**Figure 2:**
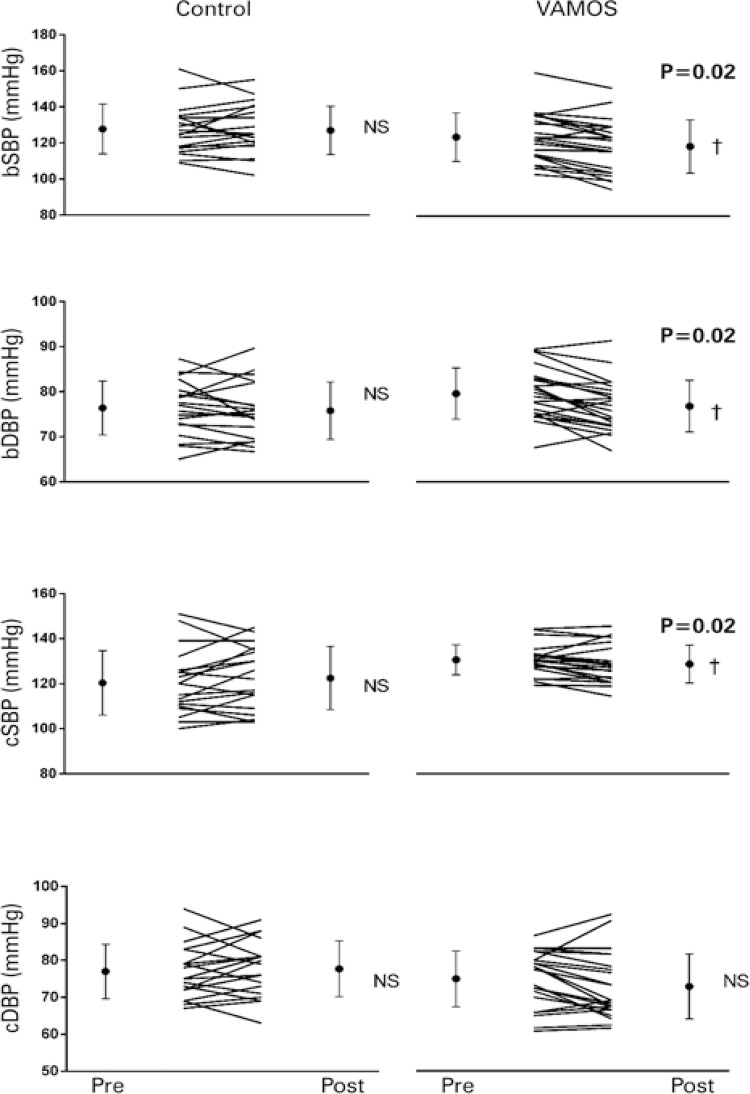
Pre- and post-intervention brachial and central blood pressure values of participants in both groups ^†^ p<0.05 *versus* pre. bSBP: brachial systolic blood pressure; NS: non-significant; VAMOS: *Vida Ativa Melhorando a Saúde* ; bDBP: brachial diastolic blood pressure; cSBP: central systolic blood pressure; cDBP: central diastolic blood pressure.

Pre- and post-intervention brachial and central BP values of participants of both groups are shown in figure 2 .

Group *versus* time interactions were detected for brachial and central systolic BP and brachial diastolic BP (p<0.05), with significant reductions in pre- compared to post-intervention BP values in the VAMOS (bSBP: 131.3±15.8mmHg to 125.1±17.3mmHg, with p<0.01; brachial diastolic BP: 78.6±8.3mmHg to 74.5±8.4mmHg, with p<0.01; central systolic BP: 123.6±16.3mmHg to 119.0±20.6mmHg, with p=0.02; central diastolic BP: 80.0±9.1mmHg to 77.6±10.5mmHg, with p>0.05) and no significant differences in the Control Group (bSBP: 127.6±13.7mmHg to 126.9±13.3mmHg; brachial diastolic BP: 76.4±6.0mmHg to 75.8±6.4mmHg; central systolic BP: 120.4±14.3mmHg to 122.5±14.0mmHg; central diastolic BP: 77.0±7.4mmHg to 77.7±7.5mmHg, with p>0.05). Mean intergroup differences in BP reduction corresponded to -5.4 (95% of confidence interval − 95%CI: -1.0- -9.9)/ -3.4 (95%CI: - 0.7- -6.2) and - 6.8 (95%CI: -0.9- -12.7)/- 3.1 (95%CI: 0.4- -6.8) mmHg for brachial and central systolic/diastolic BP, respectively.

Pre- and post-intervention resting heart rate, arterial stiffness and endothelial function parameters of participants in both groups are shown in table 3 .

**Table 3:** Pre- and post-intervention cardiovascular parameters of participants in both groups

Parameters	Control (n=19)	VAMOS (n=22)	Effects		

			Group	Time	Interaction
RHR, bpm					
Pre	64.0±10.4	66.2±10.4	0.18	0.46	0.23
Post	63.4±8.1	68.6±10.4			
ES	-0.06	0.23			
cfPWV, m/s					
Pre	9.9±1.8	10.5±2.3	0.65	0.83	0.64
Post	10.0±2.0	10.2±3.0			
ES	0.05	-0.11			
AI, %					
Pre	32.0±9.6	28.2±8.7	0.24	0.32	0.10
Post	32.7±5.9	25.1±9.4			
ES	0.09	-0.34			
FBF, mL·100 mL^−1^tissue·min^−1^					
Pre	3.0±1.1	2.7±0.8	0.96	0.16	0.32
Post	3.0±1.1	3.3±0.9			
ES	0	0.71			
PO-RH, mL·100 mL^−1^ tissue·min^−1^					
Pre	6.8±2.3	5.7±2.5	0.79	0.76	0.04
Post	5.7±2.4^*^	6.5±2.1^*^			
ES	-0.47	0.35			

Results expressed as mean±standard deviation.

^*^ p<0.05 *versus* pre.

VAMOS: *Vida Ativa Melhorando a Saúde* ; RHR: resting heart rate; ES: effect size; cfPWV: carotid-femoral pulse wave velocity; AI: augmentation index; FBF: forearm blood flow; PO-RH: post-occlusive reactive hyperemia.

Resting heart rate and arterial stiffness parameters did not differ significantly within or between groups (p>0.05). Significant group *versus* time interactions were detected for PO-RH (increase in pre- compared to post-intervention values in the VAMOS and reduction in the Control Group respectively; p<0.05).

Group *versus* time interactions remained significant for brachial diastolic BP (F=6.76; p=0.01) and PO-HR (F=7.66; p=0.01) following intention-to-treat analysis. No major effect of group *versus* time interactions on body composition and arterial stiffness parameters were detected, as in per protocol analyses.

## DISCUSSION

Lower brachial and central BP and improved PO-RH in hypertensive patients enrolled in the VAMOS program were the major findings of this study. Body composition and arterial stiffness parameters were not impacted by the program.

Brachial systolic and diastolic BP reduction in VAMOS Group participants suggests potential benefits of the VAMOS Sprogram for hypertension control. The fact that analysis of means as well as individual data of patients enrolled in the VAMOS program revealed brachial and central BP reduction supports homogeneous responses across patients. A previous study failed to detect BP profile changes in response to spontaneous increases in physical activity levels,^21^ suggesting behavior change programs combining healthy eating habits and physical activity promotion may have more robust effects on BP.

VAMOS program also reduced central systolic BP, a stronger predictor of cardiovascular events and target organ damage.^22^ Central systolic BP reduction in this study is in keeping with two recent non-controlled studies^23 , 24^ reporting lower central systolic BP values in hypertensive or non-hypertensive overweight and obese men, after 12 weeks of a lifestyle modification program. As in non-hypertensive populations, behavior change programs have positive effects of on brachial and central BP in hypertensive patients complying with behavior change programs.

Microvascular function is impaired in hypertensive patients^4^ and has been shown to be an independent predictor of cardiovascular events.^25^ Increased PO-RH (a microvascular reactivity index) in VAMOS program participants suggests lower endothelial inflammatory status^26^ or improved oxidant/antioxidant balance and higher nitric oxide bioavailability.^27^ Also, PO-RH reflects the interplay between physical (myogenic) factors and local metabolic vasodilator substances ( *e.g.,* prostaglandins, adenosine and ATP-gated potassium channels), besides nitric oxide.^28^ Blood pressure reduction in this study may be associated with all of these factors.

The VAMOS program had no significant impacts on arterial stiffness parameters. Arterial stiffness is thought to be a strong independent predictor of cardiovascular morbidity and mortality^29^ and plays a vital role in the pathophysiology of hypertension.^3^ Although this is not an universal finding,^30^ previous studies^23 , 31^ have shown that lifestyle modification programs combining physical activity and eating habits decrease cfPWV, a major indicator of arterial stiffness. However, these studies included regular exercise training programs and more rigorous dietary recommendations, suggesting regular physical training at proper levels of intensity combined with a controlled diet may be a key factor in arterial stiffness improvement.

Findings of this study are promising and significant from a clinical standpoint, given the positive effects of an educational multicomponent behavior change program on cardiovascular parameters in hypertensive patients. Minimal sample size requirements for detection of BP differences were met in the per protocol analysis; still sample size may have been too small to detect changes in variables, such as arterial stiffness or body composition parameters, with potential impacts on statistical power. The fact that dropout rates (approximately 50%) were higher in this compared to previous studies involving behavior change programs should be emphasized.^32 , 33^ This may have reflected external factors, such as intense traffic and constant lack of safety, time and money. Different from previous studies, this trial was conducted in a developing country and involved low-income patients. Strategies aimed at mitigating these barriers may increase adherence to behavior change programs implemented in large cities of developing countries.

The assessment of efficacy of the VAMOS program in patients with hypertension in this study was based on baseline and post-intervention cardiovascular outcomes. Future studies including other measurements of the same outcomes throughout the intervention period may provide more robust evidence of VAMOS efficacy and enable improved follow-up of patients dropping out over the course of the study.

Central BP, arterial stiffness, endothelial dysfunction and body composition assessment using high-end, scientifically supported techniques reinforces findings of this study. Intention-to-treat analysis and blinded study design should also be emphasized, since these procedures have been recommended for clinical trials.^34^

## CONCLUSION

*Vida Ativa Melhorando a Saúde* , a program aimed to encourage changes in physical activity levels and eating habits, was able to reduce brachial and central blood pressure, and improve microvascular reactivity in hypertensive patients. This program may therefore be an interesting strategy for non-pharmacological management of hypertension.
