# Supplementary material for: Efficacy of a behavior change program on cardiovascular parameters in patients with hypertension: a randomized controlled trial
Source: Einstein (Sao Paulo). 2020 Jan 27;18:eAO5227. doi: 10.31744/einstein_journal/2020AO5227 (PMC6986881; doi:10.31744/einstein_journal/2020AO5227)
Supplement: Translation

# Translation: Eficácia de um programa de mudança de comportamento sobre parâmetros cardiovasculares em pacientes com hipertensão arterial sistêmica: ensaio clínico controlado randomizado

**Authors:** Aline Mendes Gerage, Tânia Rosane Bertoldo Benedetti, Bruno Remígio Cavalcante, Breno Quintella Farah, Raphael Mendes Ritti-Dias

**Affiliations:** 1 Brasil Universidade Federal de Santa Catarina, Florianópolis, SC, Brasil.; 2 Brasil Universidade de Pernambuco, Recife, PE, Brasil.; 3 Brasil Universidade Federal Rural de Pernambuco, Recife, PE, Brasil.; 4 Brasil Universidade Nove de Julho, São Paulo, SP, Brasil.

**Keywords:** Hipertensão, Pressão arterial, Reabilitação cardíaca, Estilo de vida, Comportamentos relacionados com a saúde

## Abstract

**Objetivo:**

Analisar a eficácia do programa de mudança de comportamento Vida Ativa Melhorando a Saúde sobre parâmetros cardiovasculares em pacientes hipertensos.

**Métodos:**

Noventa pacientes hipertensos ≥40 anos foram aleatoriamente randomizados em dois grupos: Vida Ativa Melhorando a Saúde (n=45) e Controle (n=45). O Grupo Vida Ativa Melhorando a Saúde participou de um programa de mudança de comportamento que objetiva motivar mudanças na atividade física e nos hábitos alimentares, de acordo com a teoria sociocognitiva. O programa foi conduzido em grupos, durante 12 semanas consecutivas, em encontros semanais (~90 minutos), conduzidos por um profissional de edução física e um nutricionista. Um capítulo do material didático era discutido em cada um desses encontros. O Grupo Controle participou de uma única palestra educativa sobre mudanças de estilo de vida. Medidas de pressão arterial braquial e central, rigidez arterial, e de função endotelial foram realizadas nos momentos pré e pós-intervenção.

**Resultados:**

O Grupo Vida Ativa Melhorando a Saúde reduziu a pressão arterial sistólica braquial (de 131,3±15,8mmHg a 125,1±17,3mmHg; p<0,01) e central (de 123,6±16,3mmHg a 119,0±20,6mmHg; p=0,02) e a pressão arterial diastólica braquial (123,6±16,3mmHg a 119,0±20,6mmHg; p<0,01) e apresentou melhora na hiperemia reativa pós-oclusão (de 5,7±2,5mL·100mL^−1^a 6,5±2,1mL·100mL^−1^tecido·min^−1^; p=0,04). Não houve modificação na composição corporal, na frequência cardíaca e nem nos parâmetros de rigidez arterial em ambos os grupos (p>0,05).

**Conclusão:**

O Programa Vida Ativa Melhorando a Saúde melhorou a pressão arterial e a reatividade microvascular em pacientes hipertensos. Registro do estudo: ClinicalTrials.gov: NCT02257268

## INTRODUÇÃO

A hipertensão é uma das principais causas de doença cardiovascular^1^ e está fortemente associada à obesidade,^2^ ao aumento da rigidez arterial^3^ e à redução da reatividade microvascular.^4^ A inatividade física e as dietas pouco saudáveis são fatores de risco e prognóstico importantes para hipertensão, sendo a atividade física e os hábitos alimentares pontos centrais de abordagens baseadas na modificação do estilo de vida destinadas a pacientes hipertensos.^1 , 5 , 6^ Apesar dos benefícios do aumento dos níveis de atividade física e de uma dieta saudável para a saúde cardiovascular,^7^ apenas 30% dos pacientes hipertensos seguem as recomendações de atividade física,^8 , 9^ e menos de 10% relatam hábitos alimentares saudáveis.^10^

Programas de alteração de estilo de vida baseados em teorias de mudança comportamental são considerados estratégias promissoras para a reversão dessa situação. Em países desenvolvidos, programas similares de longo prazo (>6 meses) foram capazes de reduzir a pressão arterial (PA) sistólica e diastólica.^11^ Entretanto, dada a maior dificuldade de implementação de programas de longo prazo em cenários clínicos de países em desenvolvimento, em vista do custo elevado, a compreensão dos efeitos de programas de mudanças de comportamento de curto prazo voltados para a promoção do empoderamento e a autonomia do paciente, é importante. Nesse contexto, um programa de mudanças de comportamento de curto prazo, denominado Vida Ativa Melhorando a Saúde (VAMOS), foi recentemente desenvolvido no Brasil, a fim de promover a atividade física e os hábitos alimentares saudáveis entre a população adulta e idosa.^12^ A efetividade desse programa na manutenção da atividade física e na melhoria dos hábitos alimentares e da qualidade de vida de pacientes hipertensos foi demonstrada em estudo prévio desenvolvido pelo mesmo grupo de pesquisa.^13^

## OBJETIVO

Analisar a eficácia do programa Vida Ativa Melhorando a Saúde sobre os parâmetros cardiovasculares em pacientes com hipertensão arterial sistêmica.

## MÉTODOS

### Participantes do estudo

Este ensaio clínico não farmacológico, randomizado e controlado foi realizado em 2014, na Universidade de Pernambuco, em Recife (PE), com 90 voluntários. As estratégias de recrutamento foram publicadas na íntegra em estudo prévio.^14^

Os seguintes critérios de inclusão foram adotados: idade acima de 40 anos, diagnóstico de hipertensão,^15^ e uso de drogas anti-hipertensivas por um mínimo de 3 meses antes do estudo. Pacientes diabéticos, com diagnóstico prévio de doenças cardiovasculares (doença cardíaca isquêmica, insuficiência cardíaca, doença arterial coronariana, doença arterial periférica ou derrame), com incapacidades físicas ou envolvidos em programas regulares de atividade física não foram incluídos. Alterações do tipo ou dose de drogas anti-hipertensivas foram consideradas critérios de exclusão.

### Aleatorização

Os participantes foram aleatoriamente alocados em bloco (por um pesquisador sem envolvimento direto no recrutamento e na coleta de dados) para um dos seguintes grupos: VAMOS (n=45) e Controle (n=45). A alocação foi feita com base no sexo e na PA pré-intervenção, empregando-se tabela numérica aleatória.

### Desenho experimental

Antes do início da intervenção (fase pré-intervenção), todos os participantes foram instruídos a comparecerem ao laboratório duas vezes. Na primeira visita, foram realizadas as avaliações antropométricas, de composições corporal e da PA braquial. Na segunda visita, realizada no mesmo horário da visita anterior, foram mensurados PA braquial e central, rigidez arterial, fluxo sanguíneo basal e reatividade microvascular.

Os participantes do Grupo Controle participaram de uma palestra educativa sobre mudanças de estilo de vida, enquanto os do Grupo VAMOS participaram de um programa de mudança de comportamento de 12 semanas. Decorridas essas 12 semanas, todos os participantes foram reavaliados (fase pós-intervenção) empregando-se os mesmos procedimentos realizados anteriormente (fase pré-intervenção). Todos os pesquisadores envolvidos nas avaliações e análise de dados foram mantidos cegos quanto à aleatorização.

### O programa de mudança de comportamento Vida Ativa Melhorando a Saúde

Os indivíduos do Grupo VAMOS participaram de um programa destinado a motivar mudanças de comportamento relacionadas a um estilo de vida saudável no que se refere à atividade física e aos hábitos alimentares, segundo a teoria sociocognitiva.^16^ O programa consistiu de reuniões semanais grupais de 90 minutos de duração, agendadas ao longo de 12 semanas consecutivas e conduzidas por profissionais de educação física e nutrição. Um capítulo do material educacional (apostila) fornecido foi discutido em cada encontro.^13^ Resumidamente, cada capítulo da apostila incluía diversos tópicos relacionados a atividade física, hábitos alimentares saudáveis e estratégias de mudanças comportamentais, como definição de conceitos de atividade física e hábitos alimentares saudáveis, avaliação de fases de mudança comportamental, planejamento e implementação de rotinas de atividade física e alimentação saudável, superação de obstáculos e desafios, obtenção de apoio e monitoramento de progressos (uso de pedômetros, por exemplo), dicas de gerenciamento de estresse, construção de confiança e revisão de metas iniciais. Os participantes deveriam estar presentes em todos os encontros; em caso de ausência (até 25%), o conteúdo poderia ser reposto em outro momento.

### Mensuração da composição corporal

A gordura total e do tronco foi estimada por densitometria radiológica de dupla energia (Lunar Prodigy DXA, modelo NRL 41990, GE Lunar, Madison, WI), segundo as recomendações do fabricante. A porcentagem de gordura foi calculada dividindo-se a quantidade de gordura pelo peso do segmento analisado (tronco ou corpo inteiro).

### Aferição de parâmetros cardiovasculares

Os pacientes foram instruídos a fazer uma refeição leve, evitar atividades físicas moderadas a vigorosas por um mínimo de 24 horas e não fumar ou consumir álcool ou cafeína por um mínimo de 12 horas antes de chegar ao laboratório para aferição dos parâmetros cardiovasculares. As aferições foram realizadas após período de repouso de 10 minutos, com o paciente em decúbito dorsal, em ambiente tranquilo e com temperatura controlada.

A PA sistólica e diastólica braquial foi aferida no braço esquerdo utilizando-se um monitor oscilométrico automático (Omron HEM 742-E, Bannockburn, USA). As aferições foram realizadas em 2 dias não consecutivos e os parâmetros aferidos três vezes em cada dia,^17^ a intervalos de 1 minuto. Os valores médios de PA foram usados na análise. Todas as aferições foram realizadas pela mesma pessoa.

O fluxo sanguíneo no antebraço (FSA) foi avaliado por meio da pletismografia de oclusão venosa (Hokanson, EC6, USA), conforme descrições prévias.^18^ As aferições foram realizadas no antebraço não dominante, com o paciente em repouso e em decúbito dorsal. Um manguito foi colocado acima da mão e inflado até aproximadamente 240mmHg, a fim de interromper a circulação para o antebraço. Um segundo manguito foi colocado ao redor do braço e inflado até a PA subdiastólica (40 a 60mmHg) por 10 segundos, a cada 20 segundos. Um sensor de mercúrio foi colocado na maior circunferência do antebraço, para registrar alterações de circunferência em resposta ao influxo de sangue arterial. A magnitude de aumento da circunferência do antebraço foi empregada para estimar o FSA basal. Após essa manobra, a hiperemia reativa pós-oclusiva (HRPO) foi avaliada. O manguito posicionado no pulso, proximalmente ao local de aferição, foi insuflado até 200mmHg, e a oclusão foi mantida por 3 minutos. Em seguida, o manguito foi desinflado, e o FSA mensurado por 3 minutos, conforme descrição prévia. O FSA e a HRPO foram definidos como o valor médio de nove aferições, antes e após o período de isquemia, respectivamente.

A PA central e a rigidez arterial foram mensuradas pelo método de tonometria de aplanação (SphygmoCor, Atcor Medical, Sydney, Austrália). A PA central foi estimada empregando-se algoritmo de transferência de função validado fornecido pelo *software* SphygmoCor^®^. A velocidade da onda de pulso da carótida-femoral (cfPWV) e o índice de aumento foram empregados para avaliar a rigidez arterial e a reflexão de onda, respectivamente. A aferição desses parâmetros foi realizada pela mesma pessoa, de acordo com as diretrizes.^19^

### Análise estatística

Os dados foram armazenados e analisados por meio do programa (SPSS), versão 17.0, para Windows. A normalidade dos dados e a homogeneidade das variâncias foram investigadas empregando-se o teste de Shapiro-Wilk e o teste de Levene, respectivamente. As variáveis contínuas foram expressas como média e desvio padrão, e as variáveis categóricas, como frequência relativa. As diferenças pré-intervenção entre os grupos foram exploradas empregando-se o teste *t* independente ou o teste do χ^2^ . A análise de variância (ANOVA) de dois fatores para medidas repetidas foi empregada na comparação intra e entre grupos. Para variáveis cuja esfericidade foi violada, conforme indicado pelo teste de Mauchly, as análises foram ajustadas empregando-se a correção de Greenhouse-Geisser. Nos casos em que a razão F foi significante, o teste *post-hoc* de Fisher (LSD) foi empregado para a investigação de diferenças entre médias. O tamanho do efeito (ES) foi calculado para verificação da magnitude das diferenças, com base no d de Cohen.

Além da análise por protocolo (apenas participantes avaliados em ambas as fases do estudo, e com pelo menos 75% de presença nas reuniões de mudança de comportamento do Grupo VAMOS ou em palestras do Grupo Controle), realizou-se também análise por intenção de tratar. Para tal, todos os participantes aleatorizados que desistiram do estudo foram convidados para as reavaliações pós-intervenção. A abordagem baseada na repetição do último valor observado foi empregada em caso de dados faltantes (<10%).

O tamanho da amostra foi calculado empregando-se o software GPower (3.1.9), tomando-se a PA sistólica como desfecho principal, alfa de 95%, poder de 80% e um ES de 1,19,^20^ chegando-se ao tamanho amostral de 13 participantes por grupo.

O protocolo foi aprovado pelo Comitê de Ética local (protocolo 711.420; CAAE: 24252513.4.0000.0121) e registrado em ClinicalTrials.gov (protocolo NCT02257268). O Termo de Consentimento Informado foi assinado por todos os participantes.

## RESULTADOS

A figura 1 mostra o fluxograma dos participantes do estudo. De 216 indivíduos avaliados quanto à elegibilidade, 126 foram excluídos por não satisfazerem os critérios de inclusão, recusarem-se a participar ou por incompatibilidade de horários. Dos 45 participantes alocados por randomização para cada grupo, 48,9% do Grupo VAMOS (15 mulheres e 7 homens) e 42% do Grupo Controle (14 mulheres e 5 homens) participaram das 12 semanas de intervenção (≥75%), ou da palestra e de todas as avaliações pós-intervenção, mantendo o mesmo tipo e dose de drogas anti-hipertensivas. Durante o período de acompanhamento, foram registradas 38 desistências (20 no Grupo VAMOS e 18 no Controle) e 11 participantes foram excluídos por alteração de tipo ou dose de anti-hipertensivos (3 no Grupo VAMOS e 8 no Controle).

**Figura 1:** Fluxograma do estudo VAMOS: *Vida Ativa Melhorando a Saúde* .

As características gerais de ambos os grupos na pré-intervenção encontram-se dispostas na tabela 1 .

**Tabela 1:** Características gerais dos participantes dos grupos na fase pré-intervenção

Variáveis	Controle (n=19)	VAMOS (n=22)	Valor de p
Sexo feminino, (%)	74	68	0,70
Idade, anos	57±٩	59±10	0,50
Estado civil casado, (%)	47	68	0,36
Nível de escolaridade, anos de estudo	12±4	11±5	0,77
Número de drogas anti-hipertensivas	2,2±0,9	2,1±0,9	0,68
Diuréticos, (%)	53	64	0,48
Bloqueadores da AT1, (%)	53	59	0,68
Inibidores adrenérgicos, (%)	47	36	0,48
Bloqueadores de cálcio, (%)	37	27	0,52
IECA, (%)	26	23	0,79
Inibidor de renina, (%)	5	0	0,28

Resultados expressos como % ou média±desvio padrão.

VAMOS: Vida Ativa Melhorando a Saúde; AT1: receptores de angiotensina; IECA: inibidores da enzima conversora de angiotensina.

Não houve diferenças entre os Grupos VAMOS e Controle quanto às características demográficas e aos anti-hipertensivos usados na fase pré-intervenção (p<0,05). Em ambos os grupos, a maioria dos pacientes era mulher, casada e fazendo uso de mais de uma droga anti-hipertensiva, sendo as mais usadas os diuréticos e os bloqueadores da AT1.

No que se refere à composição corporal, não foram observados efeitos isolados ou interações entre grupo e tempo, sugerindo que tais parâmetros se mantiveram inalterados ao longo do estudo em ambos os grupos (p>0,05) ( Tabela 2 ).

**Tabela 2:** Composição corporal dos participantes em ambos os grupos nas fases pré e pós-intervenção

Variáveis	Controle (n=19)	VAMOS (n=22)	Efeitos		

			Grupo	Tempo	Interação
Peso, kg					
Pré	79,2±10,9	81,1±21,1	0,79	0,11	0,54
Pós	79,8±11,3	80,8±21,0			
ES	0,05	-0,01			
Gordura corporal, %					
Pré	42,2±8,3	41,4±6,5	0,71	0,37	0,94
Pós	41,9±7,9	41,0±6,4			
ES	-0,04	-0,06			
Gordura no tronco, %					
Pré	43,8±8,2	42,3±6,7	0,62	0,64	0,43
Pós	43,7±7,6	42,9±6,8			
ES	-0,01	0,09			
Massa muscular, kg					
Pré	42,0±7,2	44,3±11,1	0,81	0,70	0,10
Pós	44,0±7,2	43,1±12,2			
ES	0,28	-0,10			
DMO, g/cm^2^					
Pré	1,1±0,2	1,1±0,1	0,40	0,62	0,15
Pós	1,1±0,1	1,1±0,1			
ES	0	0			

Resultados expressos como média±desvio padrão.

VAMOS: Vida Ativa Melhorando a Saúde; ES: tamanho do efeito; DMO: densidade mineral óssea.

Os valores de PA braquial e central de ambos os grupos nas fases pré e pós-intervenção encontram-se ilustrados na figura 2 .

**Figura 2:** Valores de pressão arterial braquial e central de ambos os grupos nas fases pré e pós-intervenção ^†^p<0,05 comparado com pré. PASb: pressão arterial sistólica braquial; NS: não significante; VAMOS: Vida Ativa Melhorando a Saúde; PADb: pressão arterial diastólica braquial; PASc: pressão arterial sistólica central; PADc: pressão arterial diastólica central.

Observou-se interação entre grupo e tempo para os parâmetros PA sistólica braquial e central e PA diastólica braquial (p<0,05), com redução significante entre as fases pré e pós-intervenção no Grupo VAMOS (PA sistólica braquial: 131,3±15,8mmHg a 125,1±17,3mmHg, com p<0,01; PA diastólica braquial: 78,6±8,3mmHg a 74,5±8,4mmHg, com p<0,01; PA sistólica central: 123,6±16,3mmHg a 119,0±20,6mmHg, com p=0,02; PA diastólica central: 80,0±9,1mmHg; 77,6±10,5mmHg, com p>0,05) e ausência de diferenças significantes no Grupo Controle (PA sistólica braquial: 127,6±13,7mmHg a 126,9±13,3mmHg; PA diastólica braquial: 76,4±6,0mmHg a 75,8±6,4mmHg; PA sistólica central: 120,4±14,3mmHg a 122,5±14,0mmHg; PA diastólica central: 77,0±7,4mmHg a 77,7±7,5mmHg, com p>0,05). A diferença média de redução da PA entre os grupos foi de - 5,4 (intervalo de confiança de 95% − IC95%: -1,0- -9,9)/ - 3,4 (IC95%: -0,7- -6,2) e -6,8 (IC95%: -0,9- -12,7)/ -3,1 (IC95%: 0,4- -6,8) mmHg para a PA sistólica/diastólica braquial e central, respectivamente.

A tabela 3 mostra a frequência cardíaca de repouso e os parâmetros de rigidez arterial e função endotelial de ambos os grupos nas fases pré- e pós-intervenção.

**Tabela 3:** Parâmetros cardiovasculares de ambos os grupos nas fases pré- e pós-intervenção

Parâmetross	Controle (n=19)	VAMOS (n=22)	Efeitos		

			Grupo	Tempo	Interação
FCR, bpm					
Pré	64,0±10,4	66,2±10,4	0,18	0,46	0,23
Pós	63,4±8,1	68,6±10,4			
ES	-0,06	0,23			
VOPcf, m/s					
Pré	9,9±1,8	10,5±2,3	0,65	0,83	0,64
Pós	10,0±2,0	10,2±3,0			
ES	0,05	-0,11			
IA (%)					
Pré	32,0±9,6	28,2±8,7	0,24	0,32	0,10
Pós	32,7±5,9	25,1±9,4			
ES	0,09	-0,34			
FSA, mL·100 mL^−1^tecido·min^−1^					
Pré	3,0±1,1	2,7±0,8	0,96	0,16	0,32
Pós	3,0±1,1	3,3±0,9			
ES	0	0,71			
HRPO, mL·100 mL^−1^tecido·min^−1^					
Pré	6,8±2,3	5,7±2,5	0,79	0,76	0,04
Pós	5,7±2,4^*^	6,5±2,1^*^			
ES	- 0,47	0,35			

Resultados expressos como média±desvio padrão.

^*^p<0,05 comparado com pré.

VAMOS: Vida Ativa Melhorando a Saúde; FCR: frequência cardíaca de repouso; ES: tamanho do efeito; VOPcf: velocidade de onda de pulso carótida-femoral; IA: índice de aumento; FSA: fluxo sanguíneo no antebraço; HRPO: hiperemia reativa pós-oclusiva.

A frequência cardíaca de repouso e os parâmetros de rigidez arterial não se diferiram entre participantes do mesmo grupo ou entre os grupos ao longo do estudo (p>0,05). Observou-se interação significante entre grupo e tempo quanto à HRPO, com aumento no Grupo VAMOS e redução do Grupo Controle entre as fases pré e pós-intervenção (p<0,05).

As interações entre grupo e tempo se mantiveram significantes para PA diastólica braquial (F=6,76; p=0,01) e HRPO (F=7,66; p=0,01) após a análise por intenção de tratar. Assim como nas análises por protocolo, não foram detectados efeitos isolados ou interações entre grupo e tempo na composição corporal e nos parâmetros de rigidez arterial.

## DISCUSSÃO

A redução da PA braquial e central e a melhora da HRPO em pacientes hipertensos inscritos no programa VAMOS foram os principais achados deste estudo. A composição corporal e os parâmetros de rigidez arterial não foram afetados pelo programa.

A redução da PA sistólica e diastólica braquial observada no Grupo VAMOS mostra o potencial do programa no tratamento da hipertensão. Assim como a análise baseada em médias, a análise dos dados individuais mostrou que a maioria dos pacientes que seguiram o programa VAMOS apresentou redução tanto da PA braquial quanto central, indicando homogeneidade de respostas entre os pacientes. Em estudo prévio, não foram identificadas mudanças no perfil da PA em resposta a aumentos espontâneos dos níveis de atividade física,^21^ sugerindo que programas de mudanças comportamentais que combinam hábitos alimentares saudáveis e promoção da atividade física podem ter maior efeito sobre a PA.

O programa VAMOS também reduziu a PA sistólica central, um preditor mais robusto de eventos cardiovasculares e lesão em órgãos-alvo.^22^ A redução da PA sistólica central observada no presente estudo vai ao encontro de outros dois estudos não controlados recentes,^23 , 24^ que relataram reduções da PA sistólica central em homens obesos ou com sobrepeso, hipertensos ou não, após 12 semanas de participação em um programa de modificação de estilo de vida. Portanto, assim como observado em populações não hipertensas, programas de mudança de comportamento também exercem efeitos positivos sobre a PA braquial e central em pacientes hipertensos.

A função microvascular encontra-se alterada em pacientes hipertensos^4^ e é considerada preditor independente de eventos cardiovasculares.^25^ Os participantes do programa VAMOS apresentaram aumento da HRPO, um índice de reatividade microvascular. Essa melhora sugere redução do estado inflamatório endotelial^26^ ou melhora do equilíbrio oxidante/antioxidante, com maior biodisponibilidade de óxido nítrico.^27^ Além disso, a HRPO reflete a interação entre fatores físicos (miogênicos) e substâncias metabólicas vasodilatadoras locais, como prostaglandinas, adenosina e canais de potássio controlados por ATP, além do óxido nítrico.^28^ Todos esses fatores podem estar relacionados à redução da PA observada neste estudo.

O programa VAMOS não teve influência significante nos parâmetros de rigidez arterial, que é um preditor robusto de morbimortalidade por eventos cardiovasculares,^29^ além de ter papel fundamental na fisiopatologia da hipertensão.^3^ Embora esse não seja um achado universal,^30^ estudos prévios^23 , 31^ mostraram que programas de modificação de estilo de vida que combinam atividade física e hábitos alimentares reduzem a VOPcf, o principal indicador de rigidez arterial. Entretanto, tais estudos incluem programas regulares de treinamento físico e recomendações dietéticas mais estritas, sugerindo que o treinamento físico regular em níveis adequados de intensidade combinado com dieta controlada pode ser fator fundamental na melhora da rigidez arterial.

Os achados deste estudo são promissores e relevantes, do ponto de vista clínico, posto que um programa de mudança de comportamento educativo e com múltiplos componentes mostrou-se eficiente na melhora de parâmetros cardiovasculares em pacientes hipertensos. Embora o tamanho mínimo da amostra para detecção de diferenças de PA tenha sido respeitado na análise por protocolo, a amostra pode ter sido pequena para variáveis como rigidez artserial ou parâmetros de composição corporal, com potencial influência sobre o poder estatístico. Cabe notar que o índice de desistência observado neste estudo (aproximadamente 50%) foi mais alto do que o relatado em estudos prévios envolvendo programas de mudança de comportamento.^32 , 33^ Esse achado provavelmente reflete fatores externos, como trânsito intenso e falta constante de segurança, tempo e recursos financeiros, uma vez que o estudo foi realizado em um país em desenvolvimento e envolveu pacientes de baixa renda. Estratégias voltadas para a minimização de tais barreiras podem aumentar o índice de adesão a programas de mudança de comportamento implementados em metrópoles de países em desenvolvimento.

Neste estudo, a avaliação da eficácia do programa VAMOS em pacientes com hipertensão arterial sistêmica foi baseada nos desfechos cardiovasculares analisados no início do estudo e após a intervenção. Estudos futuros, incluindo outras medidas desses mesmos desfechos ao longo da intervenção, poderiam acrescentar profundidade à análise da eficácia do programa VAMOS e permitir melhor acompanhamento dos pacientes que saíram do estudo.

A avaliação da PA central, rigidez arterial, disfunção endotelial e dos parâmetros de composição corporal, por meio de técnicas de ponta e cientificamente comprovadas, reforça os achados deste estudo. Além disso, a análise por intenção de tratar e o emprego de desenho experimental cego devem ser enfatizados, uma vez que constituem procedimentos recomendados em ensaios clínicos.^34^

## CONCLUSÃO

O programa Vida Ativa Melhorando a Saúde *,* destinado a motivar a prática de atividade física e alterações de hábitos alimentares, foi eficaz na redução da pressão arterial braquial e central, e na melhora da reatividade microvascular em pacientes hipertensos, podendo constituir estratégia interessante no tratamento não farmacológico da hipertensão.
